# CBCT Evaluation of the Mandibular Foramen According to Age, Sex, and Sagittal Skeletal Class in Growing Individuals

**DOI:** 10.3390/children13081048

**Published:** 2026-08-06

**Authors:** İlhan Sengul, Mustafa Utkun, Mesut Kaygusuz, Mehmet Emin Dogan, Burcu Nur Turkoglu, Mehmet Sinan Doğan

**Affiliations:** 1 Department of Oral and Maxillofacial Surgery, Faculty of Dentistry, Harran University, Sanliurfa 63290, Türkiye; ilhansengul@harran.edu.tr (İ.S.); mustafautkun@harran.edu.tr (M.U.); 2 Department of Pediatric Dentistry, Faculty of Dentistry, Harran University, Sanliurfa 63290, Türkiye; mesutkaygusuz@harran.edu.tr; 3 Department of Dentomaxillofacial Radiology, Faculty of Dentistry, Harran University, Sanliurfa 63290, Türkiye; medogan@harran.edu.tr (M.E.D.); burcunurturkoglu99@harran.edu.tr (B.N.T.)

**Keywords:** mandibular foramen, cone-beam computed tomography, morphology, sagittal skeletal classification

## Abstract

**Highlights:**

**What are the main findings?**

• Statistically significant age-group differences were detected only for the M2 measurement, whereas no statistically significant differences according to sex or sagittal skeletal class were detected in this sample. Elliptical morphology was the most frequently observed form bilaterally.

**What are the implications of the main findings?**

• The findings provide CBCT-based anatomical information on mandibular foramen measurements and morphology in growing individuals. The clinical implications of the observed M2 differences require cautious interpretation because anesthetic and surgical outcomes were not evaluated.

**Abstract:**

**Objective**: This study aimed to describe the CBCT-based positional measurements and morphological distribution of the mandibular foramen in growing individuals and to compare the M1, M2, and M3 measurements according to chronological age group, sex, and sagittal skeletal class. **Materials and Methods**: This retrospective cross-sectional study included 120 CBCT images of individuals aged 6–16 years (mean 10.31 ± 2.33 years; 67 male, 53 female) selected from 750 archived scans. Participants were divided into three age groups (6–8, 9–12, and 13–16 years) and classified using the ANB angle (Class I, II, and III). Three measurements were assessed: M1 (foramen to occlusal plane), M2 (to posterior ramus border), and M3 (to anterior ramus border). Foramen morphology was classified into five types: circular-, elliptical-, triangular-, rectangular-, and parallelogram-shaped. The Mann–Whitney U and Kruskal–Wallis tests were used, followed by Bonferroni-adjusted pairwise comparisons where appropriate. **Results**: Interobserver ICC values ranged from 0.75 to 0.81, indicating good interobserver reliability. In the unadjusted comparisons, no statistically significant differences in M1, M2, or M3 were detected according to sex or sagittal skeletal class (all *p* > 0.05). Significant age-related differences were identified in M2 between the 6–8 and 9–12 year groups (right *p* = 0.003; left *p* = 0.021) and between the 6–8 and 13–16 year groups (right *p* < 0.001; left *p* = 0.003). Elliptical morphology was most prevalent bilaterally (right 39.2%; left 40.0%). **Conclusions**: Statistically significant age-group differences were detected only for M2, whereas no statistically significant differences according to sex or sagittal skeletal class were detected in this sample. Greater M2 values in the older age groups represent a greater absolute linear distance between the fixed mandibular foramen reference point and the posterior ramal reference line and should not be interpreted as evidence of posterior displacement. Elliptical morphology was the most frequently observed form bilaterally. These findings provide anatomical information from clinically indicated CBCT examinations but do not support routine CBCT acquisition solely for mandibular foramen localization.

## 1. Introduction

The mandibular canal and mandibular foramen are fundamental anatomical structures of the mandible, playing a critical role in the neurovascular integrity of the maxillofacial region by allowing passage of the inferior alveolar neurovascular bundle. These structures are of particular importance during inferior alveolar nerve block, dental implant placement, and orthognathic surgery [1,2]. The mandibular canal follows an S-shaped course within the mandible, and its relationship with the surrounding cortical bone varies among individuals [3]. Likewise, the position of the mandibular foramen differs considerably, contributing to unsuccessful inferior alveolar nerve block procedures. Although the mandibular foramen is generally located near the center of the ramus, its position may be influenced by age and craniofacial morphology [4,5,6]. This anatomical variability is particularly important in growing individuals because craniofacial development affects both the position and morphology of mandibular structures [7]. Recent pediatric CBCT studies have demonstrated age-related positional changes in the mandibular foramen relative to the ramus borders and the occlusal plane [8,9]. In particular, superior and posterior positional changes have been observed during the transition from mixed to permanent dentition [9]. These findings highlight the importance of considering developmental changes when evaluating mandibular anatomy and planning local anesthetic or surgical procedures in pediatric patients [8,9].

Craniofacial growth plays a fundamental role in establishing the morphological and functional relationship between the maxilla and the mandible. Therefore, accurate assessment of craniofacial growth and development is essential for orthodontic diagnosis, treatment planning, and the prediction of future skeletal changes [10,11]. Among the various diagnostic approaches, cephalometric analysis remains the most widely used method for evaluating sagittal skeletal relationships, with the ANB angle serving as one of the principal parameters for classifying maxillomandibular discrepancies [11,12]. As craniofacial growth progresses, changes in both sagittal and vertical skeletal relationships are accompanied by remodeling of the mandible, including alterations in mandibular rotation and facial proportions [11,13]. In particular, the balance between anterior and posterior facial height has been recognized as a key determinant of mandibular rotational pattern, which directly influences the sagittal relationship between the maxilla and the mandible [11,13].

Cone-beam computed tomography (CBCT) has become a valuable imaging modality for the three-dimensional evaluation of the dentomaxillofacial region, providing accurate visualization of osseous structures with substantially less distortion and superimposition than conventional two-dimensional imaging [14,15,16]. Because conventional radiographic techniques are inherently affected by superimposition, magnification, and geometric distortion, CBCT offers substantial advantages in the assessment of anatomically complex regions [16,17]. In particular, CBCT allows detailed three-dimensional assessment of the mandibular canal and mandibular foramen, enabling evaluation of their morphology and spatial relationships with adjacent anatomical landmarks using axial, coronal, sagittal, and cross-sectional reconstructions [9,18]. Recent systematic reviews have further demonstrated that CBCT provides improved three-dimensional visualization of craniofacial structures when its use is appropriately justified, particularly in selected orthodontic and dentomaxillofacial applications [16,19]. Consequently, CBCT represents a valuable imaging method for evaluating clinically and surgically relevant anatomical structures, including the inferior alveolar nerve, mandibular canal variations, and the mandibular foramen. Its ability to depict these structures may contribute to more accurate anatomical assessment and treatment planning during implant placement, orthognathic surgery, and procedures involving the inferior alveolar nerve [16,19,20].

Although several CBCT-based studies have investigated the position and morphology of the mandibular foramen in pediatric populations, most have primarily focused on age-related positional changes or descriptive anatomical characteristics of the mandibular foramen [8,9]. In addition, although sagittal skeletal relationships have been extensively evaluated in orthodontic and craniofacial research, the potential association between sagittal skeletal classification and mandibular foramen position during growth has received limited attention [11,12,13]. Moreover, few studies have simultaneously evaluated the influence of age, sex, sagittal skeletal classification, and mandibular foramen morphology using CBCT in a pediatric population, leaving an important gap in the current literature [8,9,21]. Therefore, the present study aimed to evaluate the position and morphology of the mandibular foramen using CBCT according to age, sex, and sagittal skeletal classification in growing individuals. We hypothesized that mandibular foramen measurements would differ according to developmental stage and sagittal skeletal pattern.

## 2. Materials and Methods

### 2.1. Study Design and Sample Selection

This study was designed as a retrospective cross-sectional radiological investigation. Cone-beam computed tomography (CBCT) images archived in the Department of Oral and Maxillofacial Radiology, Faculty of Dentistry, Harran University, obtained between 2022 and 2024, were evaluated. All the CBCT examinations had been obtained previously for routine clinical indications, and no CBCT scan was acquired solely for research purposes. A total of 750 CBCT images were screened; following the application of inclusion and exclusion criteria, 120 images were included in the study. Age groups were established according to chronological age and divided into three categories to facilitate the evaluation of developmental changes during growth. Although these age ranges generally correspond to the early mixed dentition, late mixed dentition, and permanent dentition periods, participants were grouped according to chronological age rather than individual dental development. This classification facilitated the evaluation of age-related changes in the position and morphology of the mandibular foramen during growth.

Inclusion criteria were defined as follows: images belonging to individuals aged 6–16 years; complete bilateral eruption of the mandibular first permanent molars; adequate image quality free of motion artifacts, distortion, or metal artifacts; clear bilateral visualization of the mandibular canal entry region; availability of age and sex data for the respective individuals; and measurability of the ANB angle to allow assessment of the sagittal skeletal relationship.

Exclusion criteria were defined as the presence of pathological lesions, cystic formations, or osseous destruction affecting the mandibular region; a history of orthodontic treatment or orthognathic surgery; and inadequate image quality or incomplete data. The study was approved by the Clinical Research Ethics Committee of Harran University (HRÜ/24.03.03) and was conducted in accordance with the principles of the Declaration of Helsinki.

### 2.2. CBCT Imaging Parameters

The CBCT images used in this study were acquired using a Castellini X-Radius Trioplus 3D unit (Imola, Italy) as part of routine clinical procedures. Standard imaging parameters were as follows: tube voltage of 70 kVp, tube current of 6.3 mA (for pediatric individuals), field of view (FOV) of 140 × 165 mm, isotropic voxel size of 0.3 mm, and slice thickness of 0.2 mm.

### 2.3. Image Analysis

All CBCT data were exported in DICOM (Digital Imaging and Communications in Medicine) format and analyzed using IRYS 2.0 imaging software (Castellini, Italy). Images were evaluated using multiplanar reconstructions in the axial, sagittal, and coronal planes, together with cross-sectional images, to allow detailed assessment of the mandibular canal and mandibular foramen region. All the evaluations were performed under standardized ambient lighting conditions using a medical-grade monitor (Advantech KT-R240FEE, Kostec, Republic of Korea). All the CBCT scans were independently evaluated by two dentomaxillofacial radiologists with 3 (B.N.T.) and 7 (M.E.D.) years of experience. For all the linear measurements, the same reproducible reference point on the superior border of the mandibular foramen was identified on the axial CBCT image and maintained throughout the multiplanar reconstruction process. For the final statistical analysis, the mean of the continuous measurements obtained by the two observers was used, whereas disagreements in the categorical morphology assessments were resolved by consensus. Inter-observer reliability was assessed using a randomly selected scan of 60 CBCTs representing 50% of the data. For continuous measurements, a two-way random effects intraclass correlation coefficient (ICC) model based on absolute agreement and mean measurements was used. ICCs were calculated using a two-way random effects model, with random effects for subjects and fixed effects for measures. The definition of absolute agreement was applied. For Single Measures, the reported values correspond to ICC (3, 1). For Average Measures, the reported values correspond to ICC (3, k), where k equals the number of items (in this case, 2). Interobserver agreement for the categorical morphology assessment was evaluated using Cohen’s kappa coefficient before consensus. Intraobserver reliability was not assessed.

### 2.4. Statistical Analysis

An a priori sample-size calculation was performed using G*Power version 3.1.9.7 (Heinrich Heine University, Düsseldorf, Germany). The calculation was conducted under the F-test family using the “ANOVA: fixed effects, omnibus, one-way” procedure as an approximation for the planned Kruskal–Wallis comparison of mandibular foramen linear measurements among three independent age groups. No single linear measurement was designated as the primary outcome for the sample-size calculation. Assuming a group size of 3, a Cohen’s f of 0.40 [22,23], a significance level of 0.05, and a statistical power of 95%, the minimum required total sample size was calculated as 102 participants. The effect size was selected as a conventional large-effect assumption because no directly comparable preliminary or published data were available for estimating the expected effect. A total of 120 participants were ultimately included.

Effect sizes were expressed as r for Mann–Whitney U tests and epsilon-squared (ε^2^) for Kruskal–Wallis tests. Continuous variables were presented as mean ± standard deviation (SD) for descriptive purposes, whereas all the inferential statistical analyses were based on non-parametric methods. Categorical variables were presented as frequencies and percentages. All the statistical tests were two-sided, and a *p*-value < 0.05 was considered statistically significant.

### 2.5. Skeletal Classification

**Figure 1:**
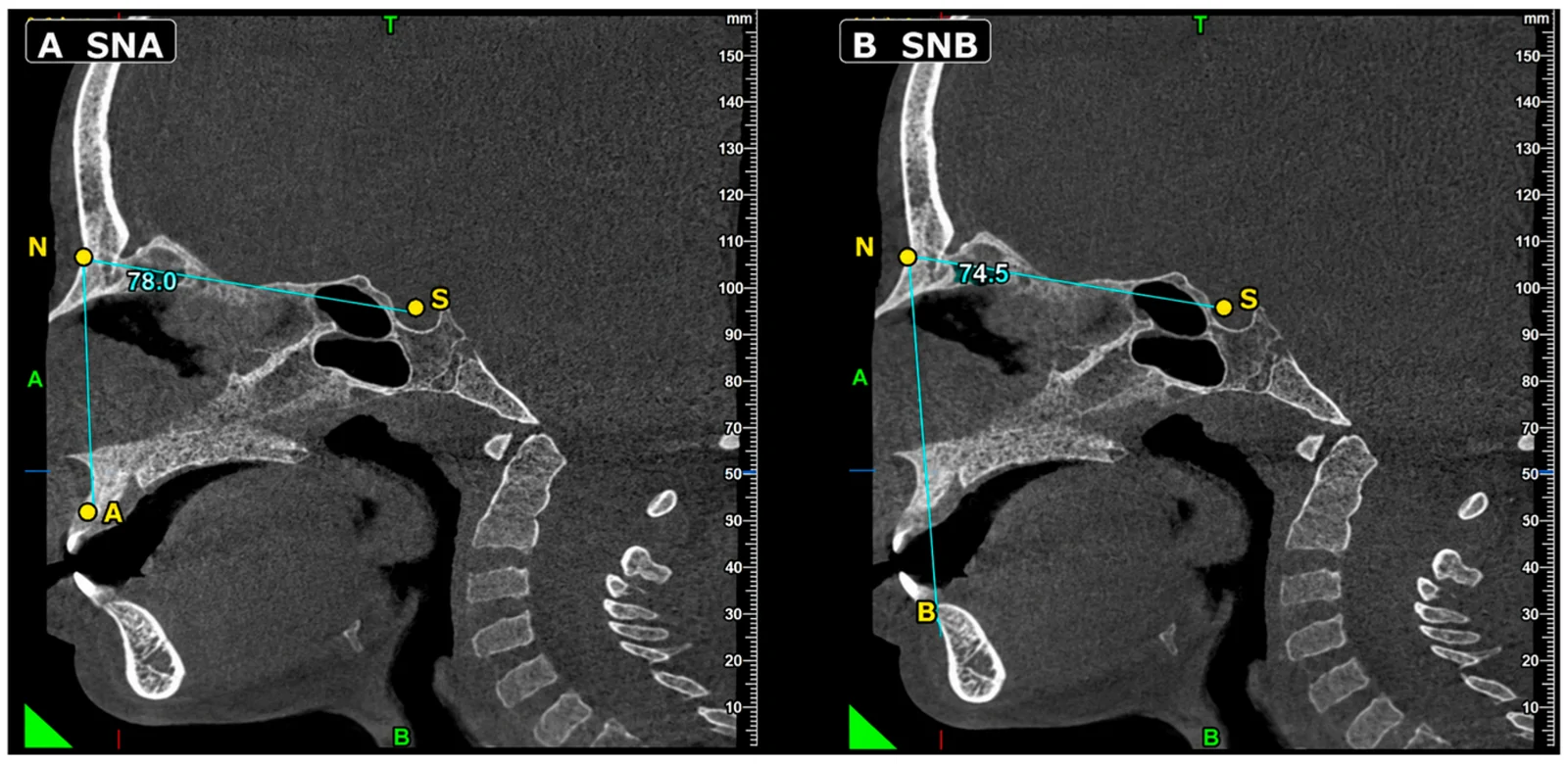
Representative sagittal CBCT sections illustrating the cephalometric measurements used for sagittal skeletal classification: (**A**) SNA angle and (**B**) SNB angle. The S, N, A, and B landmarks are indicated. The ANB angle was calculated as SNA − SNB.

Sagittal skeletal classification was determined using the ANB angle measured on cone-beam computed tomography (CBCT) images. Prior to measurement, the midline was determined by referencing craniofacial structures in the axial plane to ensure accurate orientation of the sagittal sections. Subsequently, the SNA and SNB angles were measured in the sagittal plane as illustrated in Figure 1; the ANB angle was then calculated as the arithmetic difference between these two values (ANB = SNA − SNB). Based on the resulting ANB values, individuals were classified into three skeletal categories: an ANB angle between 0° and 4° was defined as skeletal Class I, a value below 0° as skeletal Class III, and a value exceeding 4° as skeletal Class II. This classification is based on widely accepted cephalometric criteria commonly employed in the assessment of the sagittal skeletal relationship between the maxilla and mandible. In the present study, the ANB angle was used to compare mandibular foramen position among conventional sagittal skeletal classes rather than to assess intrinsic mandibular morphology or absolute mandibular dimensions [21].

### 2.6. M1 Value (Distance from the Mandibular Foramen to the Occlusal Plane, mm)

**Figure 2:**
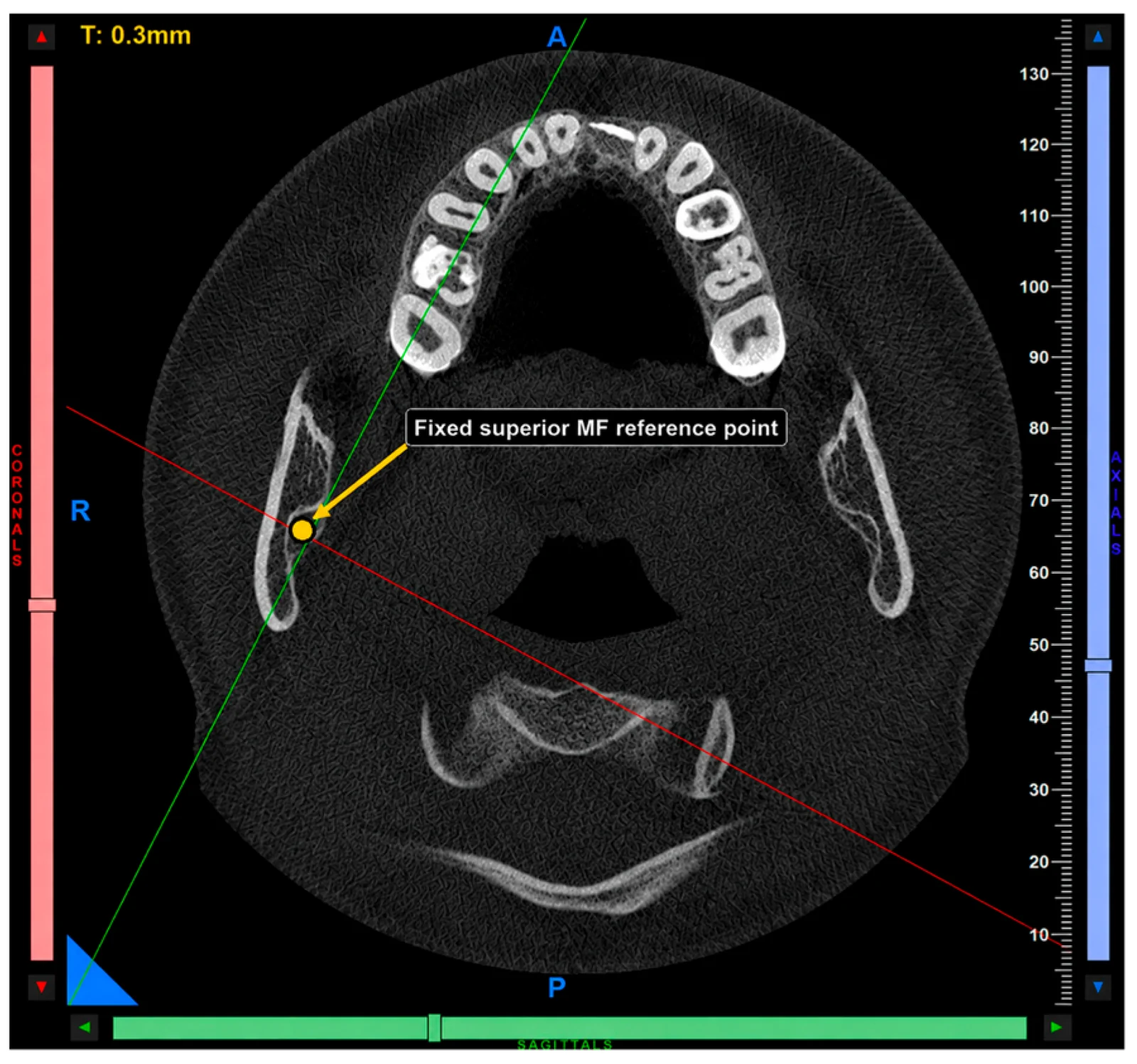
Axial CBCT image showing the fixed reference point on the superior border of the mandibular foramen (MF). This reference point was maintained throughout the multiplanar reconstruction process for the M1, M2, and M3 measurements.

**Figure 3:**
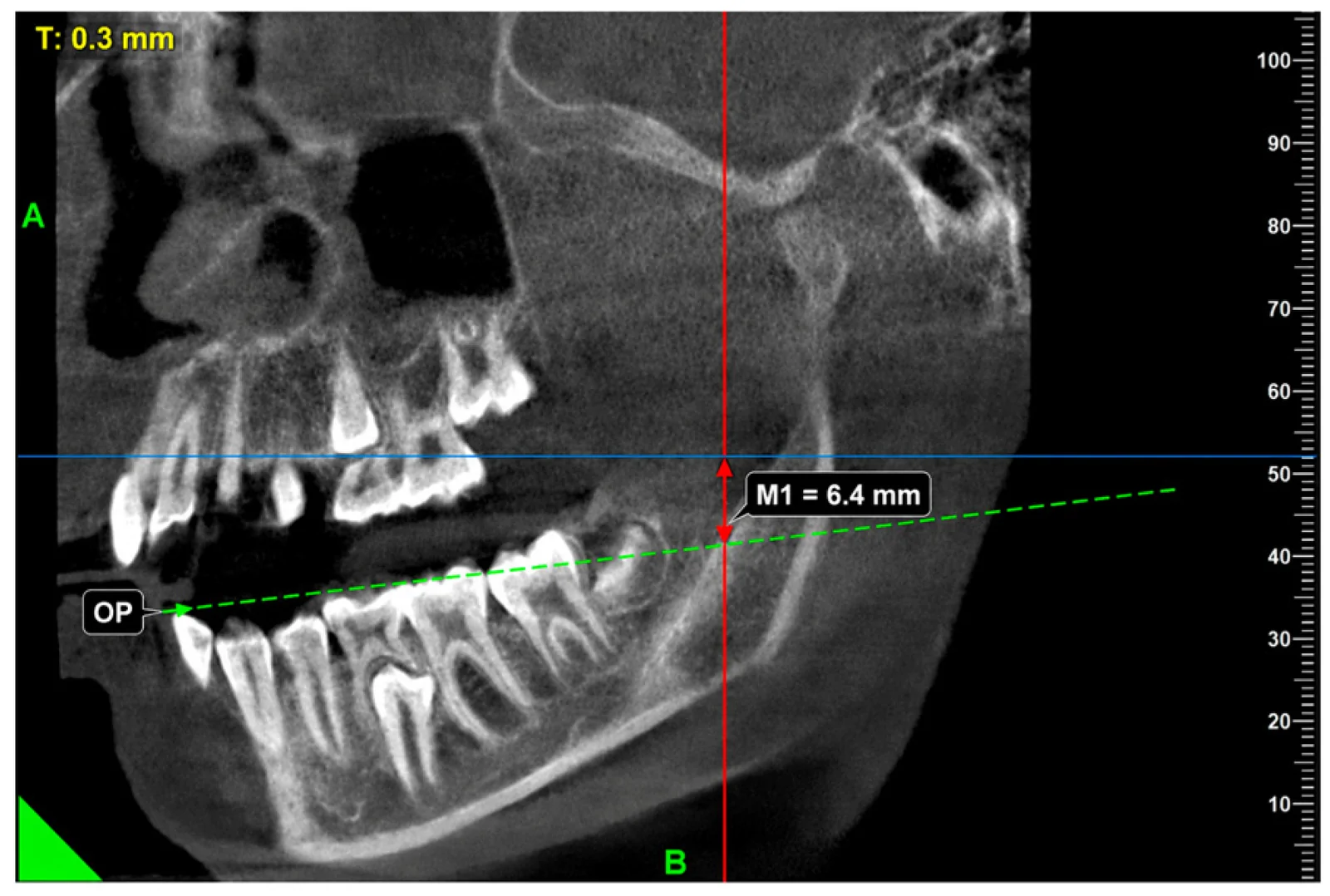
Sagittal CBCT image showing the M1 measurement (mm), defined as the shortest perpendicular distance from the fixed superior mandibular foramen reference point (MF) to the occlusal plane (OP). The occlusal plane was established by connecting the incisal edge of the mandibular canine and the distal cusp of the mandibular first permanent molar.

The distance from the mandibular foramen to the occlusal plane (M1) was evaluated using CBCT images. First, a reproducible reference point on the superior border of the mandibular foramen, corresponding to the superior aspect of the mandibular canal entrance, was identified and marked on the axial CBCT image (Figure 2). The same reference point was maintained throughout the multiplanar reconstruction process and was used for the M1, M2, and M3 measurements. The occlusal plane was established on the sagittal image by connecting the incisal edge of the mandibular canine anteriorly with the distal cusp of the mandibular first molar posteriorly. The mandibular first permanent molar was selected as the posterior reference because complete bilateral eruption of this tooth was required as an inclusion criterion, thereby ensuring a consistent occlusal reference in all the participants. Finally, the shortest perpendicular distance from the fixed superior mandibular foramen reference point to the occlusal plane was measured and recorded as M1 (Figure 3). The measurement protocol was adapted from previously published radiographic methods, with modifications to accommodate three-dimensional CBCT imaging and the anatomical landmarks used in the present study. The M1 measurements were recorded in millimeters (mm) [21,24].

### 2.7. M2 Value (Distance from the Mandibular Foramen to the Posterior Border of the Ramus, mm)

**Figure 4:**
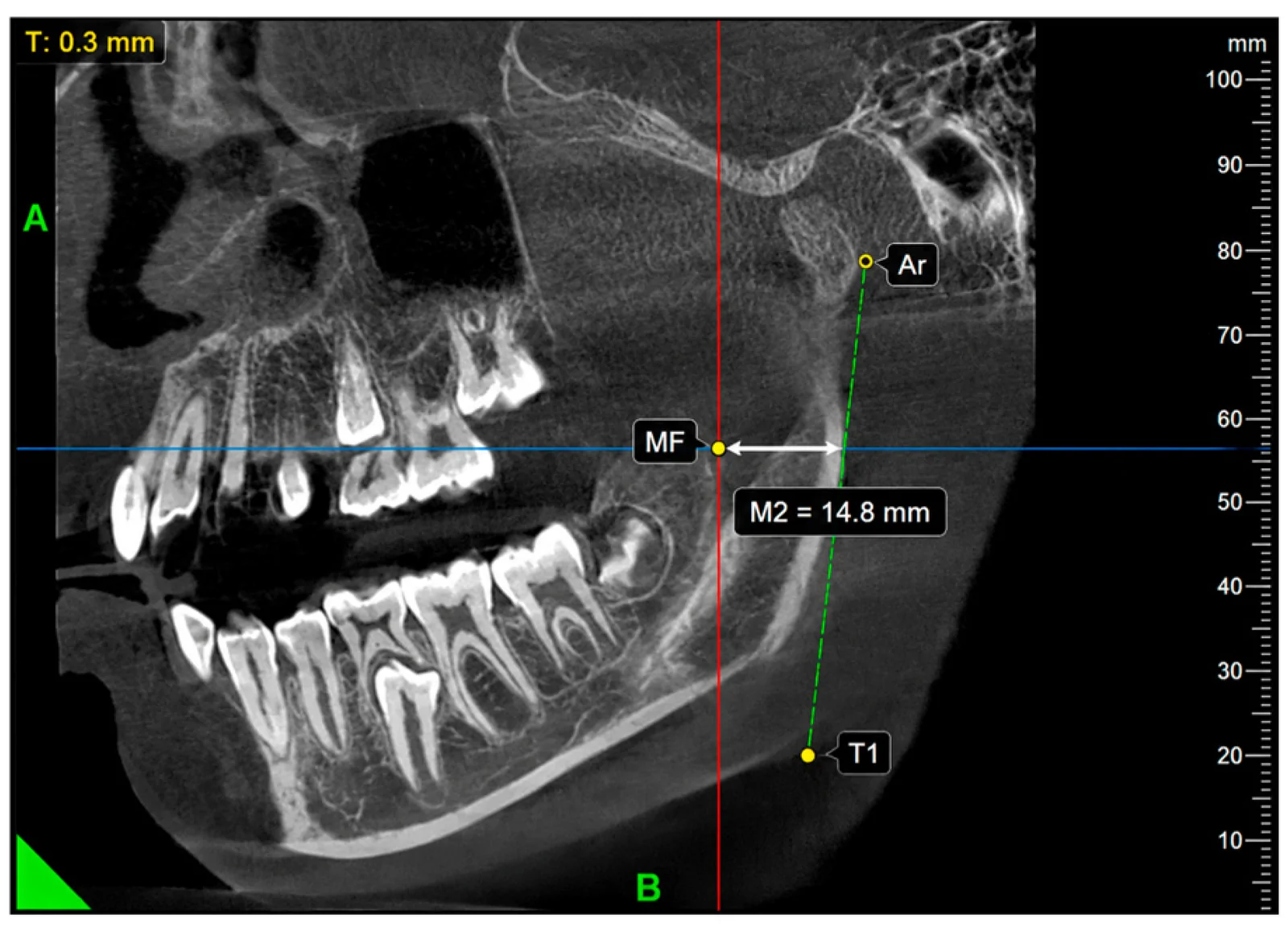
Sagittal CBCT image showing the M2 measurement (mm), defined as the shortest perpendicular distance from the fixed superior mandibular foramen reference point (MF) to the posterior ramal reference line connecting articulare (Ar) and the posterior tangent point (T1).

The distance from the fixed superior mandibular foramen reference point to the posterior ramal reference line was assessed on sagittal CBCT sections. The posterior ramal reference line was constructed by connecting the articulare (Ar) and posterior tangent point (T1). The superior mandibular foramen reference point previously identified on the axial image was maintained on the corresponding sagittal reconstruction, and the shortest perpendicular distance from this point to the Ar–T1 line was measured and recorded as M2 (Figure 4). Higher M2 values represent a greater linear distance between the fixed reference point and the Ar–T1 line and should not be interpreted as direct evidence of posterior displacement of the mandibular foramen. The measurement approach was adapted from previous CBCT-based assessments of mandibular foramen position relative to the posterior ramal border, with the Ar–T1 line used as the posterior reference in the present study. The M2 measurements were recorded in millimeters (mm) [25].

### 2.8. M3 Value (Distance from the Mandibular Foramen to the Anterior Border of the Ramus, mm)

**Figure 5:**
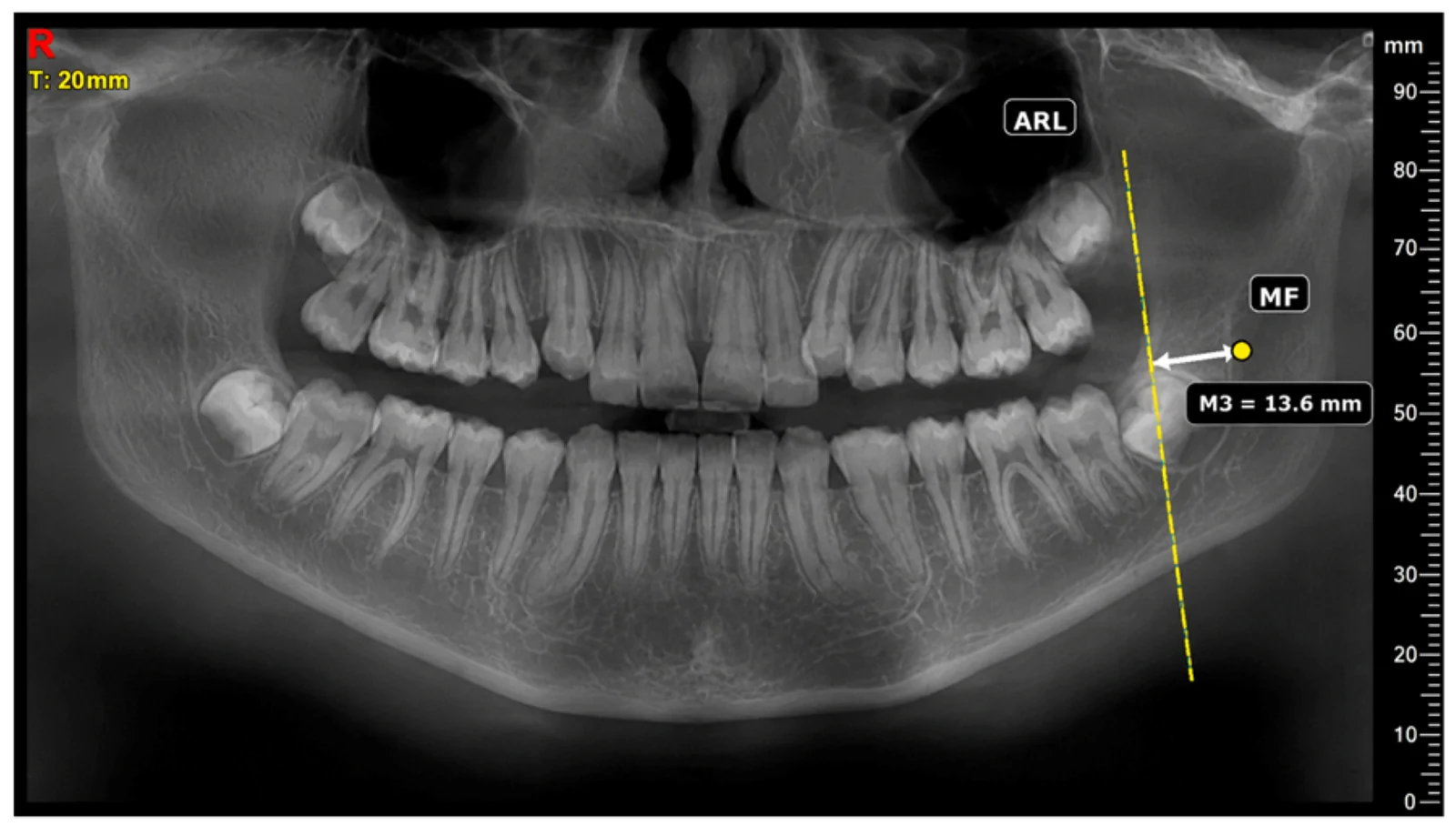
CBCT-derived panoramic reconstruction showing the M3 measurement (mm), defined as the shortest perpendicular distance from the fixed superior mandibular foramen reference point (MF) to the anterior ramal reference line (ARL).

The distance from the fixed superior mandibular foramen reference point to the anterior ramal reference line was assessed on CBCT-derived panoramic reconstructions. A panoramic reconstruction was used because the anterior border of the ramus and the mandibular foramen could not be consistently visualized on the same sagittal section owing to the three-dimensional curvature of the ramus. The reconstruction plane was manually created by tracing the mandibular arch and extending it posteriorly to include the coronoid process using the panoramic reconstruction module of IRYS 2.0 software. The anterior ramal reference line was defined as a straight tangent line placed along the anterior cortical contour of the ramus. The fixed superior mandibular foramen reference point previously identified on the axial image was projected onto the panoramic reconstruction and maintained throughout the measurement procedure. The shortest perpendicular distance from this fixed reference point to the anterior ramal reference line was measured and recorded as M3 (Figure 5). The measurement approach was adapted from previous CBCT-based assessments of mandibular foramen position relative to the anterior border of the ramus, with a standardized CBCT-derived panoramic reconstruction used in the present study. The M3 measurements were recorded in millimeters (mm) [24].

### 2.9. Mandibular Foramen Morphology

**Figure 6:**
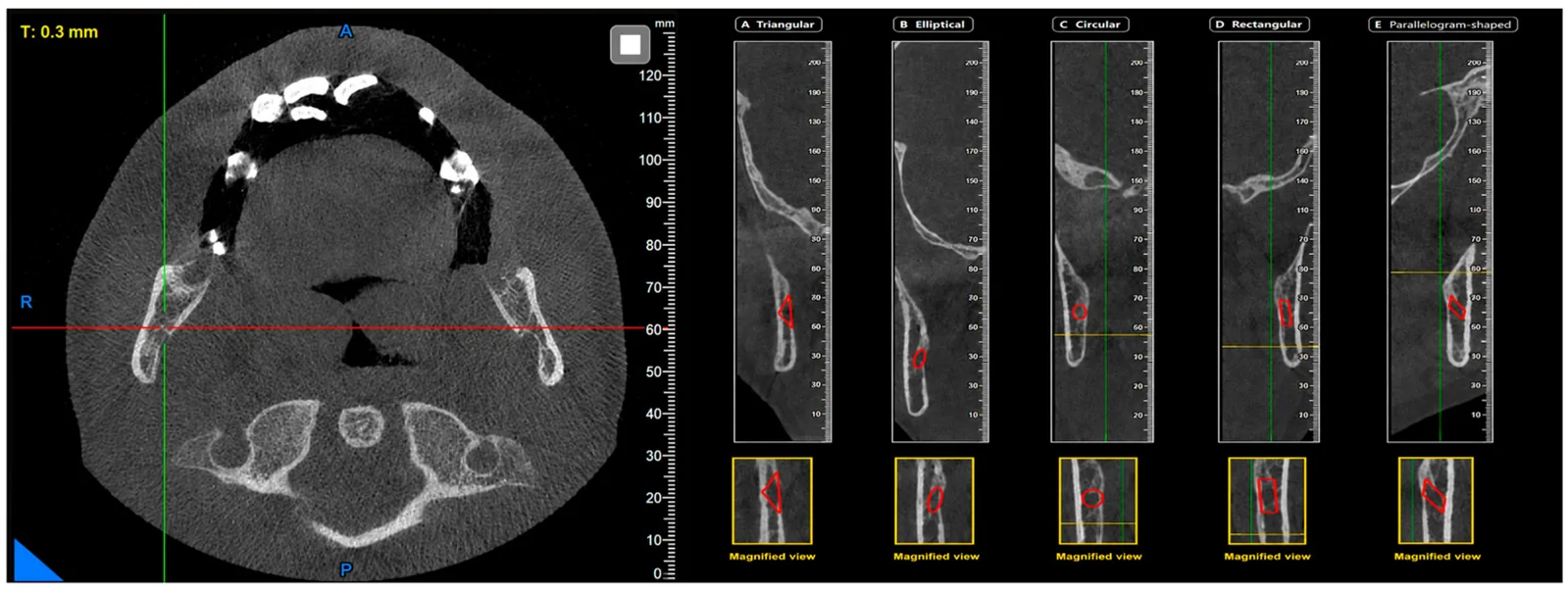
Representative CBCT images illustrating the five morphological types of the mandibular foramen: (**A**) triangular-, (**B**) elliptical-, (**C**) circular-, (**D**) rectangular-, and (**E**) parallelogram-shaped.

The morphological characteristics of the mandibular canal entry were assessed on coronal and cross-sectional planes using CBCT images. The morphological configuration of the mandibular foramen has been reported in the literature to exhibit considerable variation, and is most commonly described as V-shaped or oval in form [26]. Although various classification systems exist regarding the course and position of the mandibular canal, no standardized classification has been established for the entry morphology of the foramen [1]. For this reason, the morphological appearance of the mandibular foramen in the present study was classified into five distinct types: circular-, elliptical-, triangular-, rectangular-, and parallelogram-shaped (Figure 6). During morphological classification, the cortical boundaries and overall contour of the foramen were taken as the primary reference; assessments were subsequently verified using multiplanar reconstructions. To minimize subjectivity in morphological evaluations, all the analyses were performed independently by two experienced observers, and any discrepancies were resolved through mutual consensus. This approach is consistent with the methodological framework of previous studies in which the morphology of the mandibular canal and foramen was analyzed using three-dimensional imaging modalities.

## 3. Results

A total of 120 participants who met the inclusion criteria were included in the study. The mean age of the participants was 10.31 ± 2.33 years. The distribution of the study population according to sex, age group, and sagittal skeletal classification is presented in Table 1. Data are expressed as frequency (n) and percentage (%). Interobserver ICC values ranged from 0.985 to 1.000, indicating good reliability across the continuous measurements (Table 2). Cohen’s kappa coefficient for the categorical morphology assessment was 0.78, indicating substantial interobserver agreement before consensus.

Morphological evaluation of the mandibular foramen revealed that the elliptical type was the most frequently observed form bilaterally. On the right side, the elliptical form was followed in frequency by the circular and rectangular forms, respectively; on the left side, the elliptical form was similarly the most prevalent, succeeded by the rectangular and circular forms. The parallelogram and triangular forms were observed at comparatively lower frequencies on both sides (Table 3).

Descriptive statistics for mandibular foramen position measurements according to skeletal classification are presented in Table 4. Comparisons across groups revealed no statistically significant differences among skeletal classes with respect to the M1, M2, and M3 parameters (*p* > 0.05).

Analysis according to sex likewise revealed no statistically significant differences in any of the parameters evaluated (*p* > 0.05) (Table 5).

Descriptive statistics of the M1, M2, and M3 measurements according to age groups are presented in Table 6. Mean M2 values tended to increase with age on both the right and left sides, whereas M1 and M3 measurements showed relatively small differences among the age groups.

Multiple comparison analyses according to age group (Bonferroni adjustment) revealed statistically significant differences in the M2 parameter, which represents the linear distance between the mandibular foramen and the posterior border of the ramus (Table 7). With respect to M2 right measurements, significant differences were identified between the 6–8 and 9–12 age groups (*p* = 0.003), and between the 6–8 and 13–16 age groups (*p* < 0.001). Similarly, M2 left measurements demonstrated statistically significant differences between the 6–8 and 9–12 age groups (*p* = 0.021), and between the 6–8 and 13–16 age groups (*p* = 0.003). In contrast, no significant differences were observed among age groups in the M1 and M3 measurements (*p* > 0.05).

## 4. Discussion

The present study provides a three-dimensional assessment of mandibular foramen position and morphology in growing individuals using CBCT. Statistically significant age-group differences were detected in M2 measurements, whereas no statistically significant differences according to sex or sagittal skeletal classification were detected in this sample. The elliptical configuration was also the most frequently observed morphological type bilaterally. These findings may be clinically relevant because the mandibular foramen marks the entry of the inferior alveolar neurovascular bundle into the mandibular canal and serves as an important landmark during inferior alveolar nerve block and procedures involving the mandibular ramus [2,4]. Accurate three-dimensional characterization of its location and morphology may therefore support individualized treatment planning and reduce uncertainty when anatomical variation is expected [4]. Variation in mandibular foramen position has been identified as one of several anatomical factors that may contribute to failure of conventional inferior alveolar nerve block techniques [4,27].

The age-related differences observed in the present study are biologically plausible because mandibular growth is accompanied by continuous remodeling of the ramus, resulting in progressive changes in the relationship between anatomical landmarks. Bone deposition along the posterior border of the ramus, together with resorption along its anterior surface, contributes to alterations in ramus morphology while maintaining overall mandibular growth and function [7,10]. As condylar growth increases ramus height, remodeling of the mandibular ramus may increase the linear distance between the mandibular foramen and the posterior border of the ramus [7,28]. This developmental pattern is consistent with current concepts of craniofacial growth, which recognize continuous remodeling of the mandibular ramus throughout childhood and adolescence [7,10,28]. Accordingly, the older age groups showed greater M2 values than the 6–8-year group. This cross-sectional pattern reflects a greater linear distance between the fixed superior mandibular foramen reference point and the posterior ramal reference line but does not demonstrate longitudinal displacement of the mandibular foramen. When CBCT has already been obtained for an independent clinical indication, the observed age-group differences may contribute to the anatomical interpretation of the mandibular foramen region; however, the present study does not establish an optimal injection site or predict anesthetic success. Our findings are consistent with recent pediatric CBCT studies demonstrating age-related positional changes in the mandibular foramen relative to the occlusal plane and the borders of the mandibular ramus during growth [8,9]. Similarly, Keskin et al. [21] reported a significant association between age and mandibular foramen position in children. However, unlike their findings, no statistically significant differences in mandibular foramen localization according to sagittal skeletal classification were detected in this sample. This discrepancy may be attributable to differences in study population, age distribution, sample composition, and measurement methodology.

No statistically significant differences according to sagittal skeletal classification were detected in the present sample. This result should not be interpreted as evidence of equivalence among skeletal classes, particularly because the Class III subgroup included only six participants and the analyses were not adjusted for age. Previous studies have demonstrated that skeletal pattern influences craniofacial morphology and jaw relationships; however, these changes are not necessarily accompanied by proportional alterations in all intraosseous anatomical landmarks [12,13]. Similarly, Castro et al. [25] reported that the position of the mandibular foramen and mandibular canal was largely comparable among skeletal classes, although significant differences were identified for selected measurements, particularly in Class II and Class III individuals. Our findings are broadly consistent with these observations; however, the relative contributions of age and sagittal skeletal classification could not be determined because multivariable analyses were not performed. One possible explanation is that sagittal skeletal classification primarily reflects the anteroposterior relationship between the maxilla and mandible rather than localized remodeling of the mandibular ramus, where the mandibular foramen is located.

These findings should also be interpreted in light of the skeletal classification method used in the present study. Although the ANB angle is one of the most widely accepted cephalometric parameters for evaluating sagittal skeletal relationships, it primarily reflects the anteroposterior relationship between the maxilla and mandible rather than localized mandibular morphology or ramus remodeling [12]. Consequently, individuals classified within the same sagittal skeletal category may still exhibit considerable variation in ramus dimensions and mandibular foramen position. Furthermore, the relatively small number of Class III participants may have reduced the statistical power to detect subtle intergroup differences. This imbalance most likely reflects both the retrospective archive-based design of the study and the lower prevalence of skeletal Class III malocclusion in the pediatric population. Future investigations incorporating larger and more balanced skeletal groups together with additional three-dimensional mandibular morphometric parameters may provide a more comprehensive understanding of the relationship between skeletal pattern and the spatial relationship between the mandibular foramen and surrounding mandibular landmarks.

No significant sex-related differences were identified in mandibular foramen position in the present study. Although sexual dimorphism influences overall mandibular dimensions, these differences do not necessarily result in changes in the relative position of the mandibular foramen during the growth period. Ahn et al. [27] reported that the mandibular foramen was located more inferiorly relative to the occlusal plane in females, whereas Koivisto et al. [29] found no significant influence of sex on mandibular canal localization. The discrepancy between previous reports may be explained by differences in study populations, anatomical reference planes, imaging protocols, and age distribution. Because skeletal maturation was not assessed, it remains unclear whether the absence of statistically significant sex-related differences was related to developmental stage, sample composition, or limited statistical power.

The elliptical morphology was the most frequently observed configuration of the mandibular foramen in the present study, whereas the triangular and parallelogram types were relatively uncommon. This distribution suggests that although the mandibular foramen exhibits considerable anatomical variation, certain morphological patterns appear to occur more consistently than others. Similar variability has been described in previous anatomical and CBCT-based studies, although direct comparisons remain difficult because different investigators have used various imaging methods and classification criteria to characterize mandibular foramen morphology [4,5]. In the absence of a universally accepted classification system for mandibular foramen morphology, a simple geometry-based approach was adopted in the present study to allow consistent identification and comparison of morphological patterns. Further studies involving larger populations and standardized three-dimensional classification methods are needed to determine whether these morphological patterns are reproducible across different ethnic groups and imaging protocols.

One of the major strengths of the present study is the use of CBCT, which enables three-dimensional visualization of the mandibular foramen without the image distortion and anatomical superimposition associated with conventional two-dimensional radiography. The use of multiplanar reconstructions allowed more precise localization of anatomical landmarks and improved the consistency of linear and morphological measurements. When independently clinically justified, CBCT permits three-dimensional evaluation of the mandibular canal and mandibular foramen without the superimposition inherent to two-dimensional radiography [16,30]. This advantage should not be interpreted as support for routine CBCT acquisition, particularly in pediatric patients. The interobserver ICC values ranged from 0.75 to 0.81, indicating good measurement reliability according to the criteria proposed by Koo and Li [31].

Several limitations of the present study should be acknowledged. First, because of its retrospective cross-sectional design, changes in the position of the mandibular foramen during craniofacial growth could not be evaluated over time. In addition, participants were grouped according to chronological age rather than skeletal maturation assessed by cervical vertebral maturation, which may have introduced variability related to individual differences in growth. Second, the relatively small number of participants with skeletal Class III malocclusion rendered analyses involving this subgroup exploratory and underpowered, limiting the ability to detect subtle differences among skeletal groups. Skeletal classification was also based solely on the ANB angle; therefore, the possible influence of vertical skeletal pattern and mandibular rotation was not assessed. Furthermore, because of the limited sample size, particularly the small number of skeletal Class III participants, multivariable analyses adjusting for potential confounding variables such as age were not performed; therefore, the associations between mandibular foramen measurements, sex, and sagittal skeletal classification should be interpreted cautiously. Another limitation was the restricted availability of CBCT images in pediatric patients, as the use of CBCT must always be justified according to radiation protection principles. In addition, although M3 measurements were performed on panoramic reconstructions derived from the CBCT dataset, reconstructed panoramic images may be susceptible to reconstruction-related distortion or measurement variability. However, all the measurements were obtained using standardized reconstruction procedures while maintaining the same mandibular foramen reference point throughout the multiplanar reconstruction process. Although the standardized reconstruction protocol and good interobserver reliability may have reduced measurement variability, reconstruction-related error cannot be completely excluded. Despite these limitations, the present findings provide additional information regarding the anatomical characteristics of the mandibular foramen during the growth period. Future studies including larger cohorts, longitudinal follow-up, skeletal maturation assessment, and standardized three-dimensional evaluation protocols may further clarify the relationship between mandibular foramen measurements, craniofacial growth, and skeletal morphology. Intraobserver reliability was not assessed, which should be considered when interpreting the reproducibility of the measurements.

## 5. Conclusions

In conclusion, within the limitations of this retrospective cross-sectional study, statistically significant age-group differences were detected only for M2, whereas no statistically significant differences according to sex or sagittal skeletal class were observed in this sample. Older age groups had greater linear distances between the fixed superior mandibular foramen reference point and the posterior ramal reference line; this finding should not be interpreted as evidence of posterior displacement of the mandibular foramen. Elliptical morphology was the most frequently observed form bilaterally. These findings provide anatomical information from clinically indicated CBCT examinations but do not establish anesthetic or surgical outcomes and do not support routine CBCT acquisition solely for mandibular foramen localization. CBCT should be used only when independently clinically indicated and in accordance with radiation-protection principles.

## Figures and Tables

**Table 1:** Distribution of Participants According to Sex, Age Group, and Sagittal Skeletal Classification.

Variable	Category	n (%)
Sex	Male	67 (55.8)
	Female	53 (44.2)
Age Group (years)	6–8	28 (23.3)
	9–12	66 (55.0)
	13–16	26 (21.7)
Sagittal Skeletal Classification	Class I	51 (42.5)
	Class II	63 (52.5)
	Class III	6 (5.0)

**Table 2:** Intraclass Correlation Coefficients (ICC [3, 1] and ICC [3, 2]) for Reliability Analysis of Right and Left Measurements.

	Measurement Type	ICC	95% Confidence Interval	F	df1	df2	*p*
Right M1	Single Measures	0.999	0.999–1.000	2595.168	59	59	<0.001
	Average Measures	1.000	0.999–1.000	2595.168	59	59	<0.001
Right M2	Single Measures	0.988	0.980–0.993	167.268	59	59	<0.001
	Average Measures	0.994	0.990–0.996	167.268	59	59	<0.001
Right M3	Single Measures	0.999	0.999–1.000	2755.822	59	59	<0.001
	Average Measures	1.000	0.999–1.000	2755.822	59	59	<0.001
Left M1	Single Measures	0.999	0.998–0.999	2370.092	59	59	<0.001
	Average Measures	1.000	0.999–1.000	2370.092	59	59	<0.001
Left M2	Single Measures	0.985	0.974–0.991	135.696	59	59	<0.001
	Average Measures	0.992	0.987–0.995	135.696	59	59	<0.001
Left M3	Single Measures	0.993	0.989–0.996	321.977	59	59	<0.001
	Average Measures	0.997	0.994–0.998	321.977	59	59	<0.001

**Table 3:** Morphological Distribution of the Mandibular Foramen.

Morphology	Right n (%)	Left n (%)
Elliptical	47 (39.2%)	48 (40.0%)
Circular	39 (32.5%)	24 (20.0%)
Rectangular	19 (15.8%)	27 (22.5%)
Parallelogram	9 (7.5%)	18 (15.0%)
Triangular	6 (5.0%)	3 (2.5%)

**Table 4:** Comparison of Mandibular Foramen Measurements According to Sagittal Skeletal Classification (Mean ± SD).

Variable	Class I (n = 51)	Class II (n = 63)	Class III (n = 6)	Effect Size (ε^2^)	Kruskal–Wallis H	*p*
Right M1 (mm)	2.64 ± 3.25	1.41 ± 3.12	2.07 ± 3.64	0.011	3.319	0.190
Right M2 (mm)	13.77 ± 1.53	13.81 ± 2.23	14.73 ± 2.60	0.000	0.122	0.941
Right M3 (mm)	13.57 ± 2.22	13.57 ± 2.13	14.93 ± 3.44	0.000	0.555	0.758
Left M1 (mm)	2.45 ± 3.56	1.02 ± 3.21	3.75 ± 4.01	0.025	4.937	0.085
Left M2 (mm)	13.58 ± 1.72	13.00 ± 1.93	12.43 ± 4.63	0.014	3.684	0.158
Left M3 (mm)	13.50 ± 2.46	13.03 ± 2.54	14.00 ± 3.45	0.000	1.156	0.561

Data are presented as mean ± SD. Comparisons were performed using the Kruskal–Wallis test. Effect size was calculated as epsilon-squared (ε^2^).

**Table 5:** Comparison of Mandibular Foramen Measurements According to Sex Mean ± SD (Mean Rank).

Variable	Male (n = 67) Mean ± SD (Mean Rank)	Female (n = 53) Mean ± SD (Mean Rank)	95% CI (Lower–Upper)	Median	Min–Max	Range	IQR	Effect Size (r)	z	*p*
Right M1	1.88 ± 3.15 (59.44)	2.06 ± 3.35 (61.84)	1.11–2.65	1.40	−4–10	14	4	0.034	−0.377	0.706
Right M2	13.90 ± 1.88 (60.45)	13.77 ± 2.11 (60.57)	13.44–14.36	14.00	9–20	10	2	0.002	−0.019	0.985
Right M3	13.41 ± 2.25 (56.78)	13.93 ± 2.23 (65.21)	12.86–13.96	13.40	9–22	13	3	0.120	−1.319	0.187
Left M1	1.46 ± 3.24 (58.26)	2.15 ± 3.74 (63.33)	0.67–2.25	0.30	−9–10	19	3	0.074	−0.807	0.420
Left M2	13.25 ± 1.71 (58.39)	13.18 ± 2.44 (63.17)	12.83–13.66	13.20	10–18	8	2	0.068	−0.748	0.454
Left M3	13.07 ± 2.66 (56.73)	13.56 ± 2.41 (65.26)	12.42–13.71	12.50	7–20	13	4	0.122	−1.335	0.182

Data are presented as mean ± SD. Comparisons were performed using the Mann–Whitney U test. IQR: Interquartile Range.

**Table 6:** Mandibular Foramen Measurements According to Age Groups.

Variable	6–8 Years (n = 28)	9–12 Years (n = 66)	13–16 Years (n = 26)
Right M1 (mm)	1.49 ± 2.39	1.98 ± 3.31	2.43 ± 3.80
Right M2 (mm)	12.59 ± 1.58	13.99 ± 2.08	14.81 ± 1.37
Right M3 (mm)	14.01 ± 1.88	13.54 ± 2.43	13.48 ± 2.14
Left M1 (mm)	0.49 ± 2.48	2.27 ± 4.04	1.86 ± 2.43
Left M2 (mm)	12.15 ± 1.41	13.23 ± 2.20	14.35 ± 1.67
Left M3 (mm)	13.52 ± 2.28	13.09 ± 2.70	13.50 ± 2.49

Values are presented as mean ± SD for descriptive purposes.

**Table 7:** Pairwise Comparisons Among Age Groups.

Dependent Variable	Age Group (I)	Age Group (J)	Mean Difference (I–J)	Std. Error	*p*	95% CI (Lower)	95% CI (Upper)
Right M1	6–8	9–12	−0.021	0.019	0.781	−0.07	0.03
Right M1	6–8	13–16	−0.048	0.020	0.051	−0.10	0.00
Right M1	9–12	13–16	−0.027	0.019	0.424	−0.07	0.02
Right M2	6–8	9–12	−0.030	0.018	0.270	−0.08	0.02
Right M2	6–8	13–16	−0.060 *	0.019	0.006	−0.10	−0.02
Right M2	9–12	13–16	−0.030	0.018	0.270	−0.08	0.02
Right M3	6–8	9–12	−0.025	0.017	0.393	−0.07	0.02
Right M3	6–8	13–16	−0.055 *	0.018	0.010	−0.10	−0.01
Right M3	9–12	13–16	−0.030	0.017	0.270	−0.07	0.01
Left M1	6–8	9–12	−0.015	0.020	0.900	−0.07	0.04
Left M1	6–8	13–16	−0.040	0.021	0.180	−0.09	0.01
Left M1	9–12	13–16	−0.025	0.020	0.420	−0.07	0.02
Left M2	6–8	9–12	−0.020	0.019	0.600	−0.07	0.03
Left M2	6–8	13–16	−0.050 *	0.020	0.040	−0.10	−0.01
Left M2	9–12	13–16	−0.030	0.019	0.270	−0.08	0.02
Left M3	6–8	9–12	−0.018	0.018	0.600	−0.06	0.02
Left M3	6–8	13–16	−0.045 *	0.019	0.030	−0.09	−0.01
Left M3	9–12	13–16	−0.027	0.018	0.270	−0.07	0.02

Bonferroni Multiple Comparison Test, *. The mean difference is significant at the 0.05 level.

## Data Availability

The original contributions presented in this study are included in the article. Further inquiries can be directed to the corresponding author.

## References

[1] Kaur R., Singla R.K., Sharma R., Singla S. (2022). Localization of mandibular foramen—A comparison between dry bones and orthopantomogram. J. Med. Life.

[2] Mortazavi H., Baharvand M., Safi Y., Dalaie K., Behnaz M., Safari F. (2019). Common conditions associated with mandibular canal widening: A literature review. Imaging Sci. Dent..

[3] Iwanaga J., Matsushita Y., Decater T., Ibaragi S., Tubbs R.S. (2021). Mandibular canal vs. inferior alveolar canal: Evidence-based terminology analysis. Clin. Anat..

[4] Sembawa S.N., Baabdullah F.H., Demyati A.K., Alsaleh O., Alzahrani A.H., Alluheybi K., Ghandourah A.O. (2025). Assessing the mandibular foramen: A cone beam computed tomography (CBCT) study on location, morphology, and clinical relevance. Open Dent. J..

[5] Li X., Chen X., Zhang Z., Wang X., Han W., Kim B.S., Yan Y., Chai G., Zhang Y. (2024). Morphological and quantitative study of the inferior alveolar nerve canal in hemifacial microsomia. Sci. Rep..

[6] Okiriamu A., Butt F., Opondo F., Onyango F. (2023). Morphology and variant anatomy of the mandibular canal in a Kenyan population: A cone-beam computed tomography study. Craniomaxillofac. Res. Innov..

[7] Capote R., Preston K., Kapadia H. (2023). Craniofacial growth and development: A primer for the facial trauma surgeon. Oral Maxillofac. Surg. Clin. N. Am..

[8] Madiraju G.S., Mahabob N., Bello S.M. (2021). Morphometric analysis of mandibular foramen in Saudi children using cone-beam computed tomography. J. Pharm. Bioallied Sci..

[9] Vathariparambath N., Krishnamurthy N.H., Chikkanarasaiah N. (2022). A cone beam computed tomographic study on the location of mandibular and mental foramen in Indian pediatric population. Int. J. Clin. Pediatr. Dent..

[10] Manabe A., Ishida T., Kanda E., Ono T. (2022). Evaluation of maxillary and mandibular growth patterns with cephalometric analysis based on cervical vertebral maturation: A Japanese cross-sectional study. PLoS ONE.

[11] Knigge R.P., Hardin A.M., Middleton K.M., McNulty K.P., Oh H.S., Valiathan M., Duren D.L., Sherwood R.J. (2022). Craniofacial growth and morphology among intersecting clinical categories. Anat. Rec..

[12] Kotuła J., Kuc A.E., Lis J., Kawala B., Sarul M. (2022). New sagittal and vertical cephalometric analysis methods: A systematic review. Diagnostics.

[13] Plaza S.P., Reimpell A., Silva J., Montoya D. (2019). Relationship between skeletal Class II and Class III malocclusions with vertical skeletal pattern. Dent. Press J. Orthod..

[14] Scarfe W.C., Farman A.G. (2008). What is cone-beam CT and how does it work?. Dent. Clin. N. Am..

[15] Kau C.H., Richmond S., Palomo J.M., Hans M.G. (2005). Three-dimensional cone beam computerized tomography in orthodontics. J. Orthod..

[16] Polizzi A., Serra S., Leonardi R. (2024). Use of CBCT in orthodontics: A scoping review. J. Clin. Med..

[17] Patel S., Dawood A., Whaites E., Pitt Ford T. (2009). New dimensions in endodontic imaging: Part 1. Conventional and alternative radiographic systems. Int. Endod. J..

[18] de Oliveira-Santos C., Souza P.H.C., de Azambuja Berti-Couto S., Stinkens L., Moyaert K., Rubira-Bullen I.R.F., Jacobs R. (2012). Assessment of variations of the mandibular canal through cone beam computed tomography. Clin. Oral Investig..

[19] De Grauwe A., Ayaz I., Shujaat S., Dimitrov S., Gbadegbegnon L., Vande Vannet B., Jacobs R. (2019). CBCT in orthodontics: A systematic review on justification of CBCT in a paediatric population prior to orthodontic treatment. Eur. J. Orthod..

[20] Angelopoulos C., Thomas S.L., Hechler S., Parissis N., Hlavacek M. (2008). Comparison between digital panoramic radiography and cone-beam computed tomography for the identification of the mandibular canal as part of presurgical dental implant assessment. J. Oral Maxillofac. Surg..

[21] Keskin A., Çiçekcibaşı A.E., Açar G., Mağat G. (2024). Position of the mandibular foramen in relation to the occlusal plane in children with skeletal class malocclusion. Eur. J. Anat..

[22] Tomasi C., Bressan E., Corazza B., Mazzoleni S., Stellini E., Lith A. (2011). Reliabi-lity and reproducibility of linear mandible measurements with the use of cone-beam com-puted tomography and two object inclinations. Dentomaxillofac. Radiol..

[23] Hu X., Cheung G.S.P., Zhang Y., Sun R., Dong F. (2023). Reliability and reproduci-bility of CBCT assessment of mandibular changes before and after treatment for Class III growing patients. BMC Pediatr..

[24] Correa S., Lopes Motta R.H., Silva M.B.F., Figueroba S.R., Groppo F.C., Ramacciato J.C. (2019). Position of the mandibular foramen in different facial shapes assessed by cone-beam computed tomography—A cross-sectional retrospective study. Open Dent. J..

[25] de Castro R.W.Q., Marlière D.A.A., Haiter Neto F., Groppo F.C., Asprino L. (2024). Positions of the mandibular foramen and canal in different skeletal classes and implications for bilateral sagittal split osteotomy. J. Maxillofac. Oral Surg..

[26] dos Santos Oliveira R., Gomes Oliveira M.A., Cintra Junqueira J.L., Kühl Panzarella F. (2018). Association between the anatomy of the mandibular canal and facial types: A cone-beam computed tomography analysis. Int. J. Dent..

[27] Ahn B.S., Oh S.H., Heo C.K., Kim G.T., Choi Y.S., Hwang E.H. (2020). Cone-beam computed tomography of mandibular foramen and lingula for mandibular anesthesia. Imaging Sci. Dent..

[28] Chen S.H., Boucher N., Chung C.H., Li C. (2025). A longitudinal cone beam computed tomography study of mandibular canal changes during growth. Orthod. Craniofac. Res..

[29] Koivisto T., Chiona D., Milroy L.L., McClanahan S.B., Ahmad M., Bowles W.R. (2016). Mandibular canal location: Cone-beam computed tomography examination. J. Endod..

[30] Guzmán J., Abarca J., Navarro P., Garay I., Arnabat-Domínguez J., Betancourt P. (2024). Morphometric analysis of the mandibular canal and its anatomical variants in a Chilean subpopulation: Cone beam computed tomography study. Diagnostics.

[31] Koo T.K., Li M.Y. (2016). A guideline of selecting and reporting intraclass correlation coefficients for reliability research. J. Chiropr. Med..

